# Decoding Non-coding Variants: Recent Approaches to Studying Their Role in Gene Regulation and Human Diseases

**DOI:** 10.31083/j.fbs1601004

**Published:** 2024-03-01

**Authors:** Edwin G. Peña-Martínez, José A. Rodríguez-Martínez

**Affiliations:** 1 Department of Biology, University of Puerto Rico-Río Piedras, 00931 San Juan, Puerto Rico

**Keywords:** non-coding variants, gene regulation, transcription factors, massively parallel reporter assay, RNA processing

## Abstract

Genome-wide association studies (GWAS) have mapped over 90% of disease- and quantitative-trait-associated variants within the non-coding genome. Non-coding regulatory DNA (e.g., promoters and enhancers) and RNA (e.g., 5′ and 3′ UTRs and splice sites) are essential in regulating temporal and tissue-specific gene expressions. Non-coding variants can potentially impact the phenotype of an organism by altering the molecular recognition of the *cis*-regulatory elements, leading to gene dysregulation. However, determining causality between non-coding variants, gene regulation, and human disease has remained challenging. Experimental and computational methods have been developed to understand the molecular mechanism involved in non-coding variant interference at the transcriptional and post-transcriptional levels. This review discusses recent approaches to evaluating disease-associated single-nucleotide variants (SNVs) and determines their impact on transcription factor (TF) binding, gene expression, chromatin conformation, post-transcriptional regulation, and translation.

## 1. Non-coding Genetic Variants in Human Diseases

The haploid human genome is ~3.2 billion base pairs, with about 98% comprising non-protein-coding DNA [1–4]. Genome-wide association studies (GWAS) have revealed that over 90% of disease- and trait-associated variants have been mapped within the non-coding genome [5–9]. This raises the question: How do single-nucleotide mutations outside the protein-coding genome impact cellular and organismal phenotype? A possible reason is that the non-coding genome potentially regulates gene expression [9,10]. *Cis*-regulatory elements (CREs) are non-coding DNA sequences that regulate gene expression, including promoters, enhancers, insulators, and silencers. Promoters are near the transcription start site (TSS), where the transcriptional machinery is recruited to form the pre-initiation complex [11–13]. Enhancers are one of the most abundant CREs responsible for enhancing transcription and regulating the spatial and temporal expression of genes in a tissue-specific manner [14]. They can be located as far as megabases upstream or downstream from the target gene and have been shown to physically interact with the promoters of the target genes through protein-mediated DNA looping [12,15].

An early example of a non-coding single-nucleotide variant/polymorphism (SNV/SNP) associated with a human disease was reported in 1982 in the *β*-globin gene (*HBB*) promoter and was linked to *β*-thalassemia [16]. In 2005, it was reported that this non-coding mutation resulted in the loss of a binding site for GATA1, which interacts with other transcription factors (TFs), such as CCAAT-enhancer-binding proteins (C/EBPs) and Krueppel-like factor 1 (KLF1), to modulate *HBB* expression [7,17,18]. Advances in DNA sequencing and functional genomics assays have propelled studies on the role of non-coding variants in regulatory regions of the genome to understand human pathophysiology, genetic diagnosis, and treatments. Non-coding variants can impact cellular and organismal phenotypes by altering the molecular recognition of CREs and disrupting transcriptional and post-transcriptional regulation of gene expression [19]. This review discusses advances in identifying functional non-coding SNVs and quantifying their impact on gene regulation. We mostly focus on research in GWAS SNVs but will also highlight examples of work on non-GWAS variants and their role in human diseases.

## 2. Non-coding Variants in Transcription Factor-DNA Binding

**Fig. 1.**
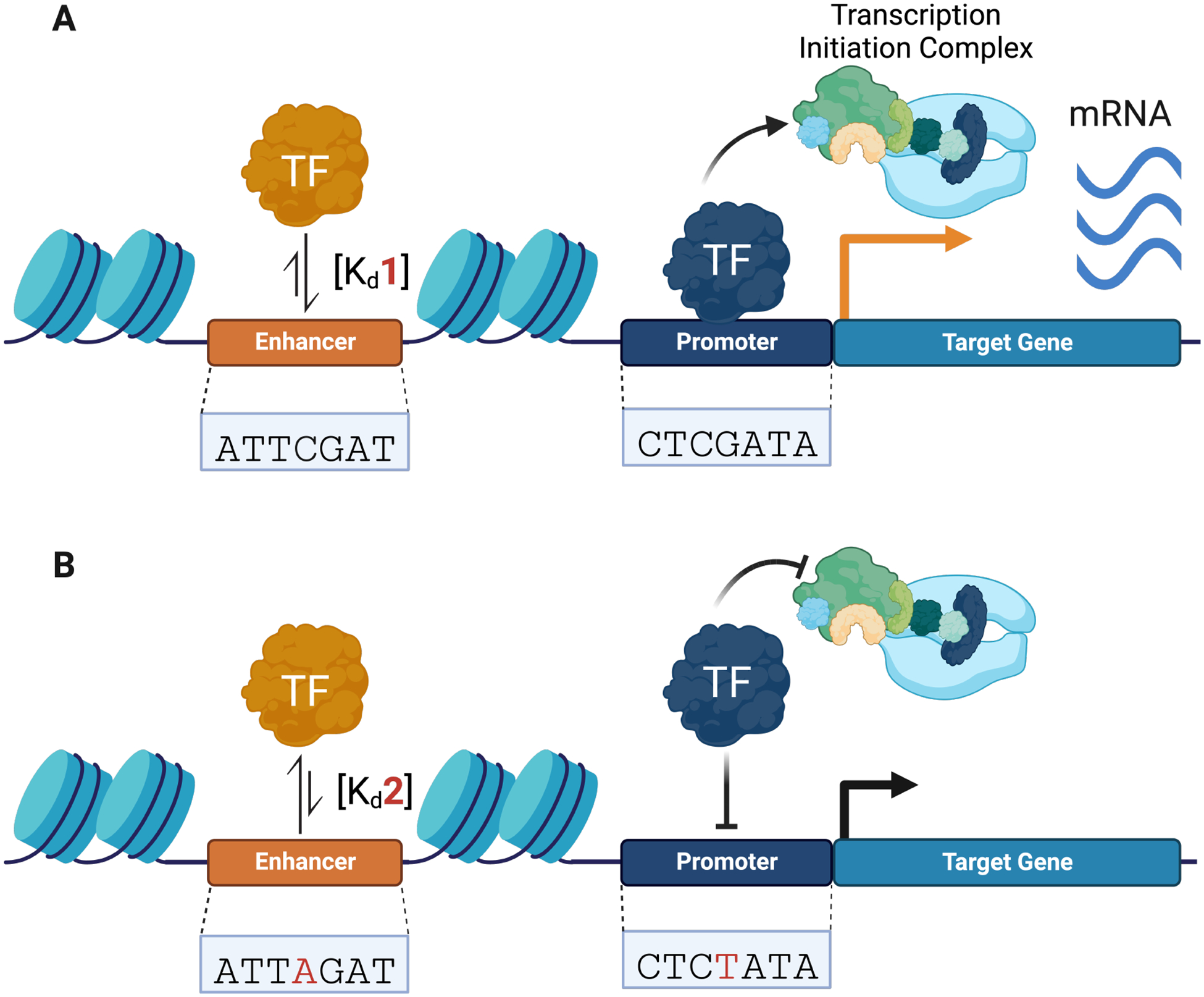
Non-coding variants can alter transcription factor (TF)–DNA binding activity, transcriptional machinery recruitment, and gene expression. (A) TFs bind to regulatory DNA (e.g., promoters and enhancers) and recruit transcriptional machinery to initiate gene expression. (B) Non-coding variants can change TF–DNA binding affinities, altering transcriptional complex recruitment and gene expression. Changes in TF–DNA binding affinities are represented by equilibrium arrows. Changes in gene expression are represented by a black (decrease) and orange (increase) arrow.

SNVs can modulate genomic binding by regulatory proteins, such as transcription factors (TFs), which are sequence-specific DNA-binding proteins that bind to CREs (e.g., promoters and enhancers) and recruit the transcriptional machinery needed to regulate gene expression (Fig. 1A) [20–23]. TFs target their specific binding sites through their DNA binding domains (DBDs), which in eukaryotes recognize short sequences of 6–12 bp [24–26]. Non-coding SNVs have been shown to alter TF-DNA recognition, leading to gene dysregulation (Fig. 1B) [6,27,28]. These variants can increase or decrease the affinity of TFs for a specific DNA sequence through the creation or disruption of TF-binding motifs [29–31].

Previous studies have determined changes in TF affinity through its binding site with *in vitro* assays, such as electrophoretic mobility shift assays (EMSA) [32]. Recently, Peña-Martínez *et al*. [33] identified five cardiovascular disease/trait-associated SNPs (rs7350789, rs61216514, rs7719885, rs747334, and rs3892630) predicted to alter the cardiac TF NKX2–5 DNA binding affinity and validated these predictions through EMSA. Although EMSA can be implemented to evaluate how non-coding SNPs can impact the formation of the TF-DNA complex and quantify changes in dissociation constant (K_*d*_), it is a low throughput method [34,35]. High-throughput methods to determine TF-DNA binding preferences [36], such as protein binding microarrays (PBMs) [37], mechanically induced trapping of molecular interactions (MITOMI) [38], systematic evolution of ligands by exponential enrichment followed by sequencing (SELEX-seq) [39,40], and bacterial and yeast one-hybrid (B1H) [41,42], have contributed a wealth of information on the intrinsic TF DNA-binding specificity.

The Fordyce lab developed microfluidic-based high-throughput approaches to determine differences in TF affinities through Binding Energy Topography by sequencing (BET-seq) [43] and simultaneous transcription factor affinity measurements via microfluidic protein arrays (STAMMP) [44]. BET-seq can estimate Gibbs free energy of binding (ΔG) for over one million DNA sequences in parallel at high energetic resolution by determining the DNA sequencing count at a TF concentration. Using BET-seq, they measured changes in binding energy for all possible combinations of 10 nucleotide flanking regions (NNNNNCACGTGNNNNN) in the yeast TFs Pho4 and Cbf1 [43]. They were able to quantify changes in binding energies as small as ~0.5 kcal/mol between flanking regions, equivalent to mutating the core motif of Pho4 and Cbf1. Using STAMMP, they can express and purify over 1500 TFs while measuring affinities in parallel by determining the occupancy of fluorescently labeled DNA (Alexa-647) and TF (GFP). Through this approach, they expressed ~210 Pho4 missense mutants and measured binding affinities for DNA sequences with substitutions along the core binding motif and the 5′/3′ flanking regions, resulting in >1800 K_*d*_ measurements in a single experiment [44].

Jung *et al*. [45] developed high-performance fluorescence anisotropy (HiP-FA), a microscopy-based fluorescence polarization method using fluorophore-labeled DNA. TF–DNA complexes have a larger molecular weight than the unbound DNA, resulting in a decreased rotational speed and increased FA. Using HiP-FA, Jung *et al*. [45] determined the DNA-binding specificity for 26 purified TF DBDs from *Drosophila* and changes in affinity for all 33 possible 1-mismatch variants in the homeobox protein Bicoid (Bcd) 11-mer consensus sequence. Bray *et al*. [46] developed the Customizable Approach to Survey Complex Assembly at DNA Elements (CASCADE), a PBM-based method to profile cofactor recruitment by TFs through antibody labeling. They used CASCADE to profile cofactor recruitment at 1712 SNPs associated with eQTLs and chromatin accessibility (caQTLs) changes that altered binding motifs for multiple ETS–family TF–cofactor complexes in myeloid cells. Through this approach, Bray *et al*. [46] found that non-coding variants also impact cofactor recruitment, which is essential in regulating gene expression. Yan *et al*. [47] developed SNP-SELEX, a high-throughput multiplexed TF–DNA binding assay, and evaluated the differential binding of 270 human TFs on 95,886 type-2 diabetes-associated SNPs (permutated to all four bases and included SNPs in linkage disequilibrium). An oligo pool was synthesized with 40 bp genomic DNA centered on the SNP and flanking regions for polymerase chain reaction (PCR) amplification and barcoding for sequencing. Using full-length TFs and DBDs, they performed six rounds of enrichment and measured 828 million TF–DNA interactions [47].

Despite the advancements in high-throughput assays to measure changes in binding affinity, the number of TF (>1600 in humans) and GWAS SNP (>500,000) combinations greatly exceeds the capacity of these techniques [8,48,49]. Many computational approaches have implemented position weight matrices (PWMs) and position frequency matrices (PFMs), which describe TF binding preferences, to identify SNVs that alter TF binding motifs. PWMs and PFMs are typically generated from *in vitro* experimental data, such as mechanically induced trapping of molecular interactions (MITOMI) [50], PBMs [37], SELEX-seq [39], and B1H [41] and from chromatin immunoprecipitation followed by sequencing (ChIP-seq) [51–53]. The development of these *in vitro* methods has led to the development of motif-based predictive models, such as SNP2TFBS [54] and atSNP [55], which use PWMs from the JASPAR [56] database to predict the impact of non-coding variants in TF binding. These predictive models can integrate variants from databases, such as the 1000 Genomes Project [57] and dbSNP [48], to make *in silico* calculations that determine the disruption or formation of a TF binding site (TFBS) compared to a reference genome [54,55]. Examples of other bioinformatics resources that aid in identifying SNPs altering TFBS are sTRAP [58], motifbreakR [59], Raven [60], rSNP-MAPPER [61], OncoCis [62], and HaploReg [63]. However, models that rely solely on PWMs may not be sufficient to predict changes in affinity accurately.

Predictions using PWMs assume nucleotides contribute to binding in an additive and independent manner but ignore sequence features such as dinucleotides, DNA shape, and complex intracellular patterns [64–66]. Nishizaki *et al*. [67] developed an SNP effect matrix pipeline (SEMpl), a computational approach that considers data of TF endogenous binding (ChIP-seq), chromatin accessibility (DNase-seq), and TF-binding patterns (PWMs) to predict intracellular-binding patterns more accurately. SEMpl significantly outperforms the traditional PWM models at predicting changes in affinity by non-coding SNPs using *in vitro* validation through EMSA [67]. However, the previously mentioned techniques are less effective at predicting tissue-specific binding events altered by non-coding variants. Boytsov *et al*. [68] recently developed ANANASTRA, an upgraded version of ADASTRA [69], a web server that can accurately predict allele-specific binding events of TFs in different cell types [68]. This program requires inputs from four databases: allele-specific binding events from GTRD (ChIP-seq data) [70], binding patterns from HOCOMOCO (TF motif predictions) [71], a list of variants from dbSNP (rs-IDs) [48], and tissue-specific context from the GTEx project (eQTL) [72].

Machine learning models, such as support vector machine (SVM) and deep learning-based convolutional neural networks (CNN), have been widely used to predict changes in TF binding due to SVMs [73–75]. VandenBosch *et al*. [76] used ATAC-seq data to train a gapped k-mer SVM (gkm-SVM) model to predict changes in TF binding to all possible SNPs on 3773 human retinal CREs. Alternatively, CNNs, such as DeepFun [77] and AgentBind [78], are deep learning-based frameworks trained with ChIP-seq and DNase-seq to accurately predict tissue and cell type-specific TF differential binding because of non-coding variants. To further predict the functionality of non-coding SNPs, Wang *et al*. [79] developed DeFine, a CNN that also implements Hi-C data to map genes affected by risk variants while quantifying real-valued TF binding intensities.

## 3. Non-coding Variants in Gene Expression

Non-coding variants can impact cellular/organismal phenotypes as a downstream effect of altering TF–DNA binding by changing gene expression and the dysregulation of gene regulatory networks (GRNs) (Fig. 1B). Gene reporter assays are a popular method for quantifying the impact of regulatory variants by measuring the promoter and enhancer activity on a reporter gene [80,81]. Jiang *et al*. [82] identified three novel regulatory SNVs from 195 conotruncal heart defect patients that impaired GATA6 binding at the promoter of *TBX1*, resulting in decreased expression as determined by a dual-luciferase reporter assay. Many of the traditional enzyme-mediated gene reporter assays, such as luciferase [83] and *β*-galactosidase [84], are effective at evaluating changes in expression caused by non-coding variants but with a low-to-medium throughput.

Massively parallel reporter assays (MPRA) are an emerging high-throughput technique that substitutes standard enzyme assays with mRNA expression detection [85]. A library of thousands of regulatory elements or genomic-variant candidates is cloned into an expression vector with unique barcodes that can be quantified through DNA and RNA sequencing to determine the gene expression fold change or through flow cytometry in the case of fluorescent proteins. Lu *et al*. [86] used MPRA to evaluate 3073 GWAS systemic lupus erythematosus (SLE)-risk variants and observed allele-dependent enhancer activity in 16% of the risk variants. Through this approach, they nominated 51 causal variants in 27 SLE-risk loci with allelic impact on gene regulation. Another high-throughput assay to measure regulatory element activity is self-transcribing active regulatory region sequencing (STARR-seq). In STARR-seq, candidate CREs are cloned downstream of a minimal promoter and an open reading frame, removing the need to use barcodes by directly sequencing the transcribed element [87]. Toropainen *et al*. [88] used a multiplex STARR-seq assay to evaluate the enhancer activity of 34,344 vascular disease trait GWAS variants and observed allele-specific enhancer activity for 5711 SNPs. For example, rs17293632:C>T was nominated as a causal variant in smooth muscle cells by creating an AP-1 motif and reducing the expression of SMAD3, a TF that has been extensively characterized in smooth muscle cells of the vascular wall [88]. Going a step further to evaluate regulatory SNVs in a developing animal has occurred through the development of a high-throughput enhancer-insertion mouse reporter assay named enSERT, which uses CRISPR/Cas9-directed mutagenesis to quantify the enhancer activity of multiple variants in developing mouse embryos through *β*-galactosidase staining. Kvon *et al*. [89] developed this method and evaluated mutations on all nucleotides of ZRS (789 bp), a limp-specific enhancer. They observed abnormal enhancer activity from 71% of previously reported polydactyly-causal variants, providing further insight into causality and molecular mechanisms [89].

Experimental MPRA datasets have been implemented to train predictive models to enhance the prediction of functional non-coding variants. Yang *et al*. [90] developed presence-only with an elastic net penalty (PO-EN), a semi-supervised model that integrates MPRA data with epigenetic features (chromatin accessibility, methylation, histone modifications, etc.) to predict the regulatory effects of genetic variants. The developers of PO-EN reported greater accuracy at identifying GWAS SNPs with differential enhancer activity in a tissue- and cell-specific manner than other deep-learning models. Dong *et al*. [91] developed Score of Unified Regulatory Feature (SURF), a computational model that incorporates MPRA data to Regulome DB [92] functional genomics features (e.g., chromatin accessibility, histone variants, and TFBS) to predict the effect of variants on gene expression. SURF was tested in the Fifth Critical Assessment of Genome Interpretation (CAGI5) regulation saturation challenge. SURF outperformed other models in predicting the effect of 17,500 SNPs in disease-associated promoters and enhancers [91]. Movva *et al*. [93] developed a CNN-based method that utilizes MPRA data to predict and interpret the transcriptional regulatory activity of non-coding variants, Deep RegulAtory GenOmic Neural Network (MPRA-DragoNN). MPRA-DragoNN successfully predicted patterns in TF activity and gene expression events affected by reduced LDL cholesterol level-associated variants from GWAS [93].

## 4. Non-coding Variants in CRE Interactions

**Fig. 2.**
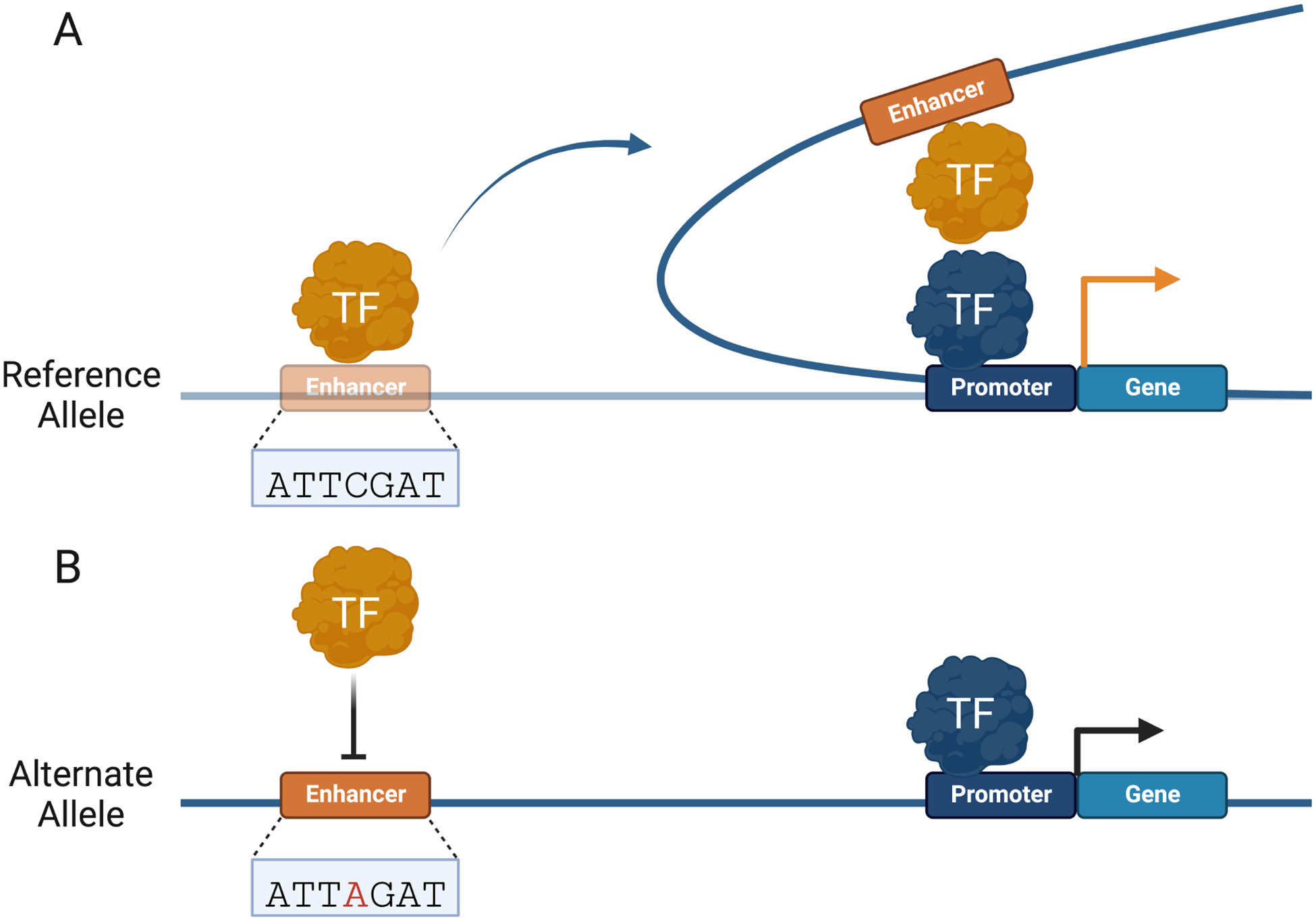
Non-coding variants can alter *Cis*-regulatory element (CRE) interactome. (A) TFs facilitate promoter–enhancer interactions by forming topologically associating domains (TADs) to regulate gene expression. (B) Non-coding variants can alter TAD boundaries and CRE interactions that regulate gene expression. Changes in gene expression are represented by an orange (increase) and black (decrease) arrow.

For over 30 years, DNA looping has been used to model how distal regulatory elements, such as enhancers, are brought near promoters to regulate gene expression (Fig. 2A) [94]. Advances in chromosome conformation capture (3C) technologies, such as circular 3C (4C) and 3C carbon copy (5C), have led to a better understanding of genome conformation, dynamics, and physical proximity between genomic elements [95–97]. These methods rely on restriction enzyme digestion of crosslinked chromatin and ligation of proximal elements to determine spatial proximity between genomic regions [98]. Coupled with massively parallel DNA sequencing, 3C assays have fueled widespread adoption and increased understanding of the genome structure on varying scales [97]. The human genome is organized in topologically associating domains (TADs), which provide an additional level of gene regulation by allowing distal CREs to interact with target promoters [99]. Understanding long-range genomic interactions is necessary to understand the potentially disruptive role of CRE variants in human diseases (Fig. 2B). High-throughput chromosome conformation capture (Hi-C) methods have proven more effective at identifying functional variants than mapping the nearest gene of GWAS single nucleotide polymorphisms (SNPs) [100]. CREs are capable of long-range interactions over one megabase (Mb) through DNA looping, skipping several genes [15,101].

Promoter-capture Hi-C (PCHi-C) measures the frequency of genome-wide promoter interactions [102]. Orlando *et al*. [103] screened 19,023 promoter fragments to identify non-coding driver SNVs that alter the colorectal cancer (CRC) cell regulatory landscape. They identified a recurrently mutated CRE that resulted in increased interactions with the *ETV1* promoter and a significant upregulation of ETV1, commonly overexpressed in CRC. Selvarajan *et al*. [104] used PCHi-C to determine the effect of genome-wide coronary artery disease (CAD)-associated non-coding SNPs within liver-specific enhancers. They identified 1277 potential CAD-causal SNPs with allele-specific regulatory activity and 621 target genes that may contribute to CAD phenotypes (compared to only 138 with eQTL analysis). They found PCHi-C to be a powerful technique for identifying target genes affected by non-coding variants, outperforming previous methods such as expression quantitative trait loci (eQTL) analysis.

Contrary to promoters, some enhancers have been shown to regulate the expression of multiple genes [105]. As such, PCHi-C has been adapted to understand how the enhancer-to-enhancer interactome is affected by genomic variations. Madsen *et al*. [106] used an enhancer-capture Hi-C (ECHi-C) capture array (library of 76,846 121nt RNA probes) to study the effects of genomic variants on human mesenchymal stem cells (hMSC) differentiation to adipocytes. Through this approach, they captured 17,235 putative active enhancers at 0, 1, and 10 days of adipocyte differentiation and observed that most eQTL variants increase enhancer interactomes. They found that the variant rs41281051: T>C is associated with increased interactions with the *LAMB1* locus and decreased LAMB1 expression in subcutaneous adipose tissue [106]. Hi-C library preparation followed by chromatin immunoprecipitation (HiChIP) provides an additional layer of regulatory information than PCHi-C by effectively mapping tissue-specific promoter–enhancer interactions in different cell types [107]. Chandra *et al*. [101] used H3K27ac (marks active enhancers) HiChIP to evaluate cell-specific and genotype-dependent effects of SNPs on various immune cell types. Most of the variants had a tissue-specific impact on the promoter–enhancer interactions, such as CD4^+^ T cells (rs8087912) and natural killer cells (rs13379920), which exhibited a significant decrease when compared to monocytes, resulting in a decreased expression of *EPB41L3* and *TM6SF1*, respectively.

There have been significant advances in experimental approaches to understanding non-coding variant effects on phenotypes. However, due to the overwhelming number of identified GWAS SNPs in the human genome (>500,000), prioritizing the variants to evaluate remains a challenge [48]. Computational approaches, such as predictive models and machine learning, can address this challenge and prioritize functional non-coding variants for validation. Meng *et al*. [108] used Hi-C data from human embryonic stem cells (hESC) to develop a deep learning model (DeepHiC) to predict the impact of SNPs on long-range chromatin interactions. Using ~8 million non-coding SNPs from the 1000 Genomes Project [57], they were able to successfully identify five osteoporosis-associated functional variants (rs9533090, rs9594738, rs8001611, rs9533094, and rs9533095) in an eQTL of *TNSFS11* [108]. Computational approaches have also been developed to identify cell-specific functions of non-coding variants. Yu *et al*. [109] developed a Single-Nucleus Analysis Pipeline for Hi-C (SnapHiC) to analyze 3471 neuropsychiatric disorder-associated SNPs. They observed different interactions for the same variants in different prefrontal cortical cells. For example, two enhancers containing Alzheimer’s-associated SNPs (rs112481437 and rs138137383) resulted in astrocyte-specific loops to the *APOE* gene TSS [109]. Other computational approaches have constructed gene regulatory networks (GRNs) of GWAS SNPs from 3C techniques (i.e., Hi-C and ChIA-PET) to predict causal risk variants [110]. Gao *et al*. [111] developed the Annotation of Regulatory Variants using Integrated Networks (ARVIN) and identified over 1000 risk variants for seven autoimmune diseases using disease-relevant GRNs for known causal SNPs. Using ARVIN, they successfully predicted an average of 160 risk SNPs with a significant overlap of the eQTL analysis [111].

## 5. Non-coding Variants in Post-transcriptional Regulation

**Fig. 3.**
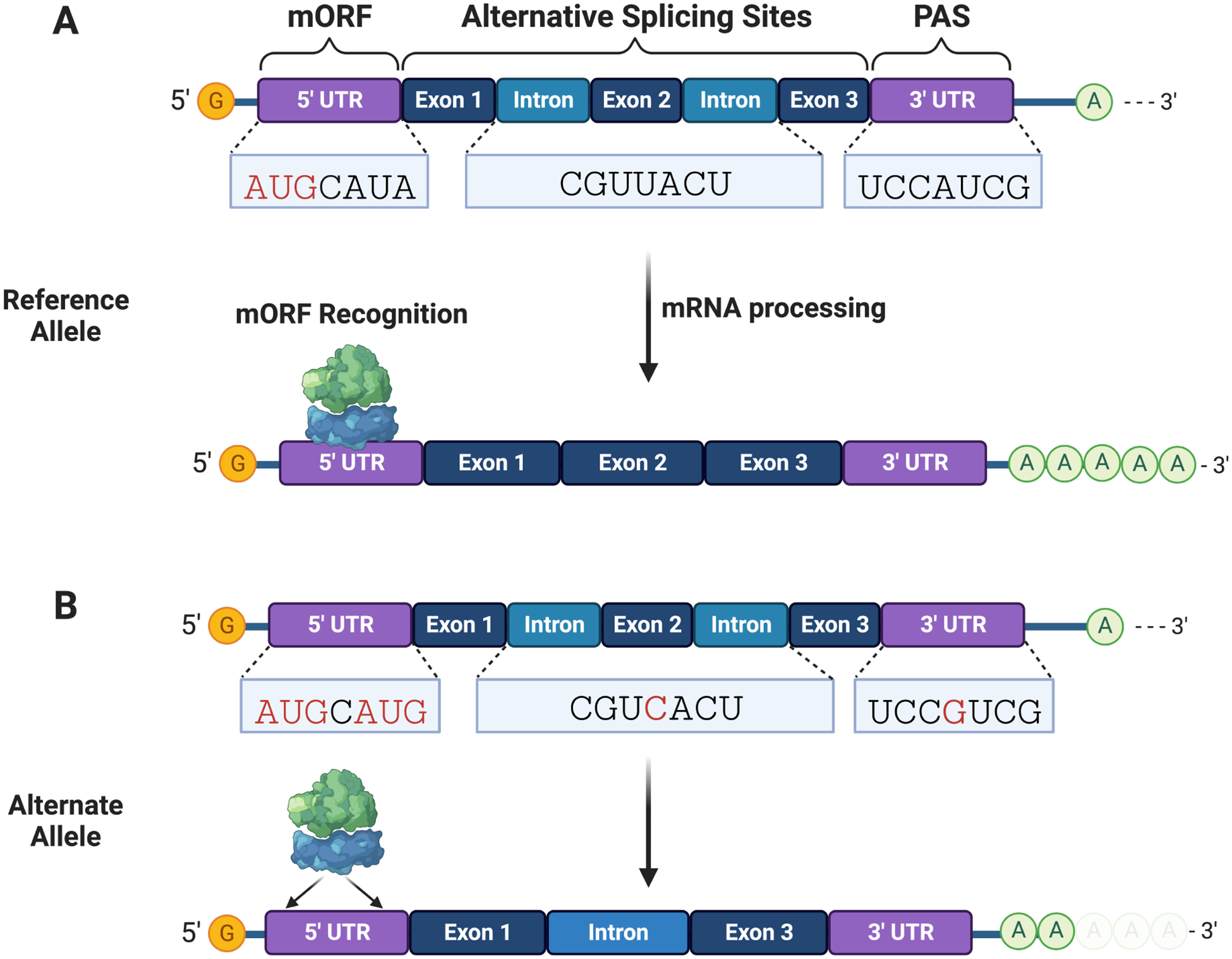
Non-coding variants can disrupt mRNA processing and translation initiation. (A) mRNA interactions with RNA-binding proteins and ribosomes are needed for processing (e.g., splicing and adenylation) and translation initiation, respectively. (B) Non-coding variants can alter splice and polyadenylation sites needed for stable mRNA processing and expression of functional protein isoforms. mRNA variants can create translation sites that compete with the main open reading frame (mORF). PAS, polyadenylation sites.

Non-coding variants can occur within the 5′ and 3′ untranslated regions (UTRs) and introns, impeding potentially altering mRNA processing (e.g., splicing, polyadenylation and cleavage, and ribosome binding and assembly) (Fig. 3A,B). Non-coding SNVs can change the binding affinity between RNA-binding proteins (RBPs) and pre-mRNA, impacting on phenotypes through post-transcriptional dysregulation [112]. Krooss *et al*. [113] described the pathomechanism of a non-GWAS SNP found in four families with moderate to severe hemophilia B. The variant created a U1snRNP binding site in the 3′ UTR region of the coagulation factor 9 (*F9*) mRNA (c.2545A>G). The binding of U1snRNP inhibited polyadenylation and proper 3′-end processing, which resulted in mRNA degradation and reduced expression of *F9* [113]. Bauwens *et al*. [114] identified eight non-GWAS variants in a group of German and Belgian patients diagnosed with *ABCA4*-associated diseases. The variants that occurred within *ABCA4* introns 2, 7, 21, 30, and 36 resulted in eight pathogenic splice variants determined by minigene splicing assays, a method that clones variant sequences into expression vectors and identifies them through reverse transcription polymerase chain reaction (RT-PCR) [114]. However, both gene expression and splicing present tissue-and cell-specific patterns, making it challenging to detect functional variants. Bronstein *et al*. [115] implemented whole-genome sequencing (WGS) and RNA-seq alongside patient-induced pluripotent stem cell (iPSC) transcriptome analysis to detect tissue-specific splicing patterns caused by non-coding variants. They cultured iPSC-derived retinal organoids from a family with inherited retinal degenerations and used RNA-seq to identify a novel pathogenic splice variant (chr8:g.87618576G>A) in the *CNGB3* gene caused by an intronic SNV [115]. WGS and iPSC from pedigrees provided an innovative alternative for the functional analysis of genomic variants where no prior knowledge or association had been established.

Variants within the 5′ UTR of a gene can affect protein translation by interfering with ribosome scanning and assembly. Zhou *et al*. [116] screened 14 genetically undiagnosed Saethre–Chotzen syndrome (SCS) patients and identified the first (non-GWAS) SCS-associated non-coding SNV (c.−263C>A and c.−255G>A) within *TWIST1*. These variants created translation start sites within the 5′ UTR of the *TWIST1* mRNA, which decreased translation of the main open reading frame (mORF), causing a more than 75% reduction in TWIST1, as determined by gene reporter assays [116]. Lim *et al*. [117] developed Pooled full-length UTR Multiplex Assay on Gene Expression (PLUMAGE), a high-throughput method that clones a luciferase gene and barcode downstream of the 5′ UTR variant to quantify mRNA transcription and translation efficiency in parallel. Using PLUMAGE on tissues from prostate cancer patients, they identified 326 mutations within the 5′ UTRs, of which 35% (114/326) was associated with altered transcription and translation [117]. Griesemer *et al*. [118] developed a Massively Parallel Reporter Assay for the 3′ UTR (MPRAu), a high throughput approach to quantify allelic expression imbalances in 3′ UTR variants in a cell-specific manner [118]. Through this approach, they tested 12,173 3′ UTR variants and identified 2368 variants that altered transcription levels across six cell types (HEK293, HEPG2, HMEC, K562, GM12878, and SK-N-SH).

With the overwhelming number of non-coding variants, computational approaches have been developed to identify and prioritize functional variants that occur in mRNA untranslated regions. Chen *et al*. [119] developed a computational pipeline coupled with experimental validation to identify functional variants within polyadenylation sites (PAS). By implementing four resources of human polyadenylation maps and two disease-associated databases, they identified 68 pathogenic variants within PAS that were validated using a modified luciferase reporter vector (mpCHECK2) designed to evaluate polyadenylation in gene expression [119]. Paggi *et al*. [120] developed a deep learning-based computational method to predict mRNA splicing points known as the Long Short-term memory network Branchpoint Retriever (LaBranchoR). LaBranchoR predictions identified 106 pathogenic variants affecting mRNA splicing, showing a substantial overlap of pathogenic variants from ClinVar and the Human Gene Mutation Database (HGMD) [120]. In contrast, Sample *et al*. [121] developed Optimus 5-Prime, a CNN trained on data from polysome profiling and RNA-seq, to predict the effect of 5′ UTR variants on ribosomal loading. They were able to predict ribosome loading for over 40,000 variants and were able to identify 45 functional disease-associated SNPs in the 5′ UTR [121].

## 6. Future Directions and Author Recommendations

Technological advances and reduced costs in DNA sequencing have resulted in an ever-increasing number of disease/trait-associated variants. This has resulted in a need to develop innovative computational and experimental strategies to determine the role and causal mechanisms of non-coding variants in human diseases and quantitative traits. The first challenge is to select or prioritize from the existing GWAS variants (>500,000). Our group and others have implemented computational approaches to prioritize variants based on a particular disease, gene target, or protein of interest (TFs or RBPs) [33,47,86,103]. We recommend incorporating multi-omics and functional genomics datasets (genomic, transcriptomic, epigenomic, etc.), which can improve the predictive power of the computational models to identify variants with a temporal- or tissue-specific impact [68,91,111,121,122]. In our previous work on cardiac TFs, we implemented predictive models (PWM- and SVM-based) to prioritize cardiovascular disease (CVD)-associated SNVs from the GWAS catalog [33,75]. Since our work has focused on CVD-associated SNVs, we have trained our predictive models with cardiac TF ChIP-seq data from human-induced cardiomyocytes (hiPSC-CM). We have also prioritized genomic variants mapped in regions active in cardiac tissue or during heart development by incorporating ChIP-seq and DNase I hypersensitivity genomic footprints (DGF) from cardiac tissue. Our recommendation and most strategies reviewed here rely on mining public databases or previous knowledge. When these options are unavailable, pedigree WGS combined with patient-derived iPSCs and transcriptomics of differentiated cells provides an alternative to identify de novo variants in specific cases [113–115,123–126].

This manuscript aimed to discuss the vast advancements in functional assays to identify causal variants for multiple human diseases and propel collaborations to describe their complete genetic mechanisms. In the future, we believe that these computational and experimental methods will be combined to achieve a genome-wide understanding of the role of SNV in human diseases. For instance, 97% of congenital heart disease (CHD)-associated variants have been mapped within the non-coding genome, including intronic, intergenic, UTRs, and regulatory regions [127–132]. Elucidating the genome-wide impact of these non-coding variants in complex biological systems, from human cardiomyocytes to CHD patients, will require a combination of methods to assay all levels of genetic regulation. Thus, a combined analysis of high-throughput technology is required to understand the impact of CHD-associated SNVs on chromatin structure (e.g., HiChIP [101]), TF–DNA and TF–cofactor interactions (e.g., CASCADE [46] and SNP-SELEX [47]), gene expression (e.g., MPRA [86] and STARR-seq [88]), RNA processing (e.g., MPRAu [118]), and translation (e.g., PLUMAGE [117]). The findings generated by such an integrative approach can produce crucial data needed to train effective models, which prioritize the functional impact of genomic variants that can be scaled to multiple diseases. Going further, knowing the causal mechanism of pathogenic SNVs is crucial for treating or even curing diseases through gene editing by CRISPR-based methods [133–135].

## 7. Concluding Remarks

Recent advancements have allowed us to understand and identify functional non-coding variants that can play a role in human diseases. Although these mutations occur outside the protein-coding genome, they can impact on phenotype by altering how regulatory proteins, such as TFs and RBP, interact with CREs and dysregulate gene expression. Non-coding variants can impact different stages of gene regulation by affecting (i) chromatin interactions (promoter and enhancer interactomes), (ii) TF affinity for their binding sites, (iii) transcriptional activity of target genes, (iv) post-transcriptional regulation (mRNA stability and splicing), and (v) translation initiation (ribosome recognition).

New methods have been developed to perform high-throughput functional evaluations of variants to determine causal mechanisms linked to human diseases (Table 1, Ref. [43–47,82,86,88,89,101,103,104,106,113–118]). Changes in chromatin interaction maps, TF–DNA binding affinity, gene expression, and translation efficiency provide evidence to support the role of many disease-associated variants. However, with the overwhelming and increasing number of variants in the non-coding genome, identifying functional variants remains challenging. Experimental data has been implemented to design computational approaches to predict and identify functional pathogenic variants. Computational pipelines and machine learning tools (SVMs and CNNs) can decipher tissue- and cell-specific patterns to predict variants with functional activity and prioritize *in vitro* validation (Table 2, Ref. [55,67,68,74,77–79,90,91,93,108,109,111,119–121]).

Despite all the progress in understanding the role of disease-associated variants within the non-coding regulatory genome, determining causality remains challenging. We hypothesize that the number of regulatory variants will continue to increase significantly while the molecular mechanisms of most reported variants remain unknown. The increased throughput and ability to functionally validate disease-associated non-coding variants will contribute to the rapid development of diagnostic methods and treatments for these diseases.

## Figures and Tables

**Table 1.** Summary of experimental methods to identify non-coding functional variants.

	Method	Throughput	Detection	Cell- and tissue-specific	Experiment	Ref
CRE-interactome	PCHi-C	High	Promoter-CRE interactome, target gene	Yes	*In vivo* (cell line)	[103,104]
	ECHi-C	High	Enhancer-CRE interactome	Yes	*In vivo* (cell line)	[106]
	HiChIP	High	Cell-type CRE interactome	Yes	*In vivo* (cell line)	[101]
TF–DNA binding	BET-seq	High	Binding free energy	No	*In vitro*	[43]
	STAMMP	High	Binding affinity	No	*In vitro*	[44]
	HiP-FA	High	Binding affinity and specificity	No	*In vitro*	[45]
	CASCADE	High	Cofactor recruitment by TFs	Yes	*In vivo* (cell line)	[46]
	SNP-SELEX	High	Binding affinity	No	*In vitro*	[47]
Gene expression	Luciferase reporter assay	Low	Bioluminescence	Yes	*In vivo* (cell line)	[82]
	MPRA	High	RNA-seq/flow cytometry	Yes	*In vivo* (cell line)	[86]
	STARR-seq	High	RNA-seq	Yes	*In vitro* (cell)	[88]
	enSERT	High	lacZ staining	Yes	*In vivo*	[89]
Post-transcriptional regulation	Luciferase reporter assay	Low	Bioluminescence	No	*In vivo*	[116]
		Low	Bioluminescence	No	*In vitro*	[113]
	Minigene splicing assays	Low	RNA-seq	No	*In vitro* (from patients)	[114]
	Patient iPSC WGS	High	RNA-seq	Yes	*In vivo*	[115]
	MPRAu	High	RNA-seq	Yes	*In vitro* (cells)	[118]
	Plumage	High	RNA-seq and bioluminescence	Yes	*In vitro*	[117]

PCHi-C, promoter-capture Hi-C; ECHi-C, enhancer-capture Hi-C; HiChIP, Hi-C library preparation followed by chromatin immunoprecipitation; BET-seq, Binding Energy Topography by sequencing; STAMMP, simultaneous transcription factor affinity measurements via microfluidic protein arrays; HiP-FA, high-performance fluorescence anisotropy; CASCADE, Customizable Approach to Survey Complex Assembly at DNA Elements; MPRA, massively parallel reporter assays; STARR-seq, self-transcribing active regulatory region sequencing; iPSC, induced pluripotent stem cell; WGS, whole-genome sequencing; MPRAu, Massively Parallel Reporter Assay for 3′ UTR.

**Table 2.** Summary of computational methods to predict non-coding functional variants.

	Program	Type	Training data	Prediction	Cell- and tissue-specific	Ref
CRE interactions	DeepHiC	Deep learning	Hi-C	Long-range chromatin interactions	Yes	[108]
	SnapHiC	Computational pipeline	Hi-C	CRE interactions	Yes	[109]
	Arvin	Network-based predictive model	Hi-C, ChIA-PET	GRNs	Yes	[111]
TF–DNA binding	atSNP	Motif-based predictive model	PWMs	TF binding	No	[55]
	SEMpl	Computational pipeline	ChIP-seq, DNase-seq, PWMs	TF binding	No	[67]
	ANANASTRA	Computational pipeline	ChIP-seq, PWMs, rs-IDs, eQTL	TF binding	Yes	[68]
	deltaSVM	SVM	ATAC-seq	TF binding	Yes	[74]
	DeepFun/AgentBind	Deep neural networks	ChIP-seq, DNase-seq	TF binding	Yes	[77,78]
	DeFine	CNN	ChIP-seq, Hi-C	TF binding, mapped gene	Yes	[79]
Gene expression	PO-EN	Semi-supervised model	MPRA	Enhancer activity	Yes	[90]
	SURF	Deep learning	DNase-seq, ChIP-seq, MPRA	Gene expression, TF binding	Yes	[91]
	MPRA-DragoNN	CNN	MPRA	Gene expression	Yes	[93]
Post-transcriptional regulation	Variant PAS Pipeline	Computational pipeline	Polyadenylation maps	PAS variants	No	[119]
	LaBranchoR	Deep learning	Splicing branchpoints	mRNA splicing points	No	[120]
	Optimus 5-prime	CNN	Polysome profiling, RNA-seq	Ribosome loading, gene expression	No	[121]

SnapHiC, Single-Nucleus Analysis Pipeline for Hi-C; SEMpl, SNP effect matrix pipeline; PO-EN, presence-only with elastic net penalty; SURF, Score of Unified Regulatory Feature; PAS, polyadenylation sites; SVM, support vector machine; CNN, convolutional neural networks; PWMs, position weight matrices.
